# Combined TDDFT and AIM Insights into Photoinduced Excited State Intramolecular Proton Transfer (ESIPT) Mechanism in Hydroxyl- and Amino-Anthraquinone Solution

**DOI:** 10.1038/s41598-017-14094-5

**Published:** 2017-10-23

**Authors:** Daoyuan Zheng, Mingzhen Zhang, Guangjiu Zhao

**Affiliations:** 1 State Key Laboratory of Molecular Reaction Dynamics, Dalian Institute of Chemical Physics, Chinese Academy of Sciences, Dalian, 116023 China; 2 Tianjin Key Laboratory of Molecular Optoelectronic Science, Institute of Chemistry, Department of Chemistry, School of Science, Tianjin University, Tianjin, 300072 China; 3 University of the Chinese Academy of Sciences, Chinese Academy of Sciences, Beijing, 100049 China

## Abstract

Time-dependent density functional theory (TDDFT) and atoms in molecules (AIM) theory are combined to study the photoinduced excited state intramolecular proton transfer (ESIPT) dynamics for eight anthraquinones (AQs) derivatives in solution. The calculated absorption and emission spectra are consistent with the available experimental data, verifying the suitability of the theory selected. The systems with the excited-state exothermic proton transfer, such as 1-HAQ, 1,5-DHAQ and TFAQ, emit completely from transfer structure (T), while the reactions for those without ESIPT including 1,4-DHAQ and AAAQ appear to be endothermic. Three reaction properties of three systems (1,8-DHAQ, DCAQ and CAAQ) are between the exothermic and endothermic, sensitive to the solvent. Energy scanning shows that 1,4-DHAQ and AAAQ exhibit the higher ESIPT energy barriers compared to 1-HAQ, 1,5-DHAQ and TFAQ with the “barrierless” ESIPT process. The ESIPT process is facilitated by the strengthening of hydrogen bonds in excited state. With AIM theory, it is observed that the change in electrons density ρ(r) and potential energy density V(r) at BCP position between ground state and excited state are crucial factors to quantitatively elucidate the ESIPT.

## Introduction

Due to the significance in modern photophysics, photochemistry and biochemistry, such as Green Fluorescent Protein (GFP)^1,2^, organic light emitting diodes (OLEDs)^3–5^ and fluorescent chemosensors^6^, the excited state intramolecular proton transfer (ESIPT) phenomenon have attracted numerous experimental and computational interests^7–16^. Researches of recent decades have shown that the molecules with ESIPT properties exist in the enol form in the ground state, stabilized by the intramolecular hydrogen bonding interactions. Upon photoexcitation, the molecules experience an ultrafast intramolecular proton transfer, giving rise to an excited state keto tautomeric form. The equilibrium between these two forms lead to various intriguing fluorescence properties for the molecules, including dual emission spectra, double proton transfer and back ESIPT process^15,17–23^.

**Figure 1:**
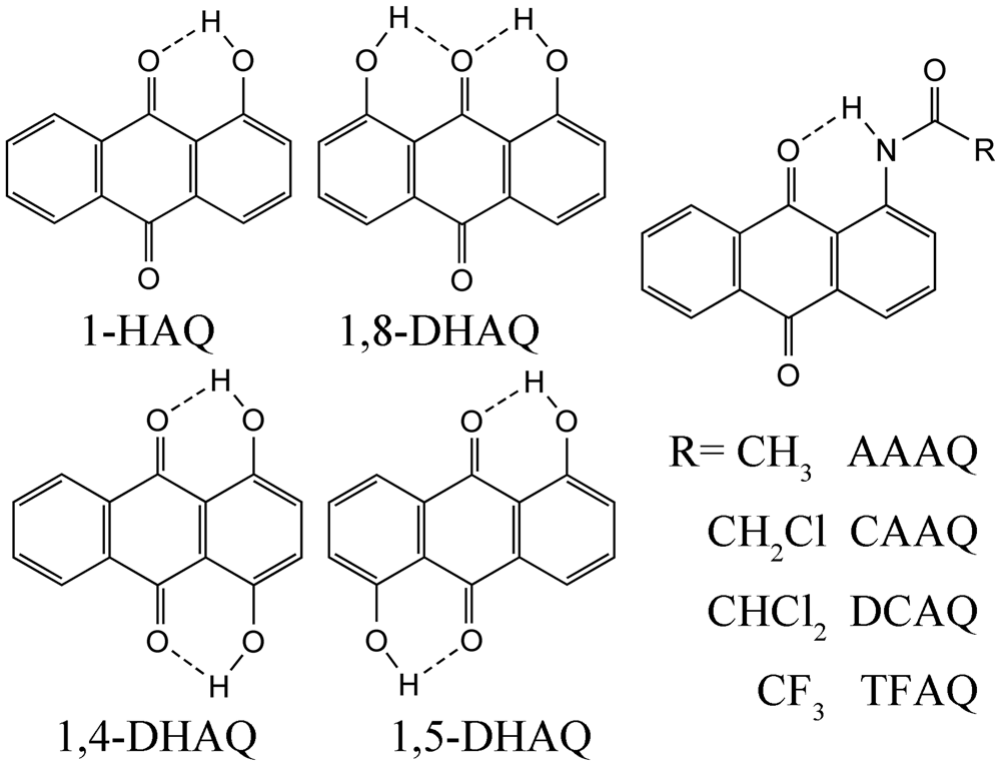
Molecular structures of HAQ, DHAQs and AYAAQs related in this work.

The anthraquinone (AQs) derivatives (Fig. 1), including hydroxyl/dihydroxy-anthraquinone (HAQ/DHAQ) and 1-(acylamino)-anthraquinones (AYAAQs)^17,18,24–28^, exhibit the unique ESIPT properties, thus presenting emerging applications in fluorescence probes, dyes and drugs^29–33^. The experimental ESIPT evidences have been confirmed for 1-HAQ^34–37^, 1,5-DHAQ^37–39^, DCAQ and TFAQ^40–43^. However, 1,4-DHAQ^37,38^ and AAAQ^40–43^ with the similar molecular structures don’t show the ESIPT progress. More interestingly, 1,8-DHAQ^25,37–39,44–47^ and CAAQ^40–43^ switch their properties in different solvents. Blank *et al*. found that the long wavelength emissions (LWE) corresponding to the tautomeric (T) in excited state were dominant for DCAQ and TFAQ in dichloromethane solvent. Only short wavelength emissions (SWE) were observed for HPAQ which the R group is n-heptyl. The emission of CAAQ was mainly SWE and slight LWE^43^. When the solvent was changed to ethanol, LWE in CAAQ and DCAQ decreased greatly^40^. The substituent effect on ESIPT for AQs have been explained by nodal-plane model proposed in 1996^48^, which have also been widely explored and proved to be a reliable theory to study the ESIPT systems^49–51^. However, this model only qualitatively describes ESPIT process based on the molecular skeletons and functional groups, without the quantitative descriptions of the dual fluorescence distribution and energy barriers, limiting an accurate understanding of ESIPT process.

In this work, the DFT/TDDFT methods were used to investigate two series of AQs in both ground and excited states. Analysis of the hydrogen bonding interactions, electronic transition energies and infrared vibrations provides the atomic insight into their ESIPT processes. The structures in ground state (S_0_(T)) were not stable with the evidence of optimized potential energy surface (PES) except for compound 1,4-DHAQ in which both the single and double protons transfer structures are stable. The AAAQ presented the unstable structure upon ESIPT, consistent with experiments^40,43^. Other seven compounds showed the stable structures at both S_1_(N) and S_1_(T), resulting in various ESIPT properties. The PES in excited state confirmed the exothermic reaction and “barrierless” ESIPT process in 1-HAQ, 1,5-DHAQ, DCAQ and TFAQ with the dominant LWE in dichloromethane. The ESIPT processes for other four compounds are endothermic with different energy barriers. 1,4-DHAQ has the highest energy barrier in S_1_ state, similar to AAAQ. The alterable ESIPT properties of 1,8-DHAQ, CAAQ and DCAQ came from their medium energy barriers. Hydrogen bond strengthening in excited state is confirmed by the redshift of vibration frequencies. AIM theory was used to investigate the relationship between ESIPT progress and property of BCP. The changes of electrons density ρ(r) and potential energy density V(r) at BCP position in ground state and excited state are significant for the ESIPT process. More importantly, the V(r) at BCP position is better than ρ(r) as a reference for hydrogen bond dynamics.

### Theoretical methods

The calculations in this work were performed by the DFT/TDDFT method^52–55^ with the Becke’s three parameter hybrid exchange functional with Lee–Yang–Parr gradient-corrected correlation (B3LYP) functional^56–58^ and 6–311 + G(d, p) basis set^59,60^ by Gaussian 09 program^61^. The graph of FMO isosurfaces is drawn by *Chemcraft* ^62^. The vertical transition energies and geometric optimization in the excited state were calculated by TDDFT method. Vibrational frequencies of both ground and excited state were computed to ensure that the geometries indeed correspond to a minimum confirmed by no imaginary frequencies. The conductor-like polarizable continuum model (CPCM) was employed to describe the implicit solvent effect (dichloromethane)^63,64^. The PES of the S_0_ and S_1_ states were calculated to explore the transfer barrier and thermodynamics effect, revealing ESPIT mechanisms.

The atoms in molecules (AIM) theory proposed by Bader is used for analyzing property of wave function and other real space functions^65–67^. The bond critical point (BCP) generally appears between attractive atom pairs, which property is closely related to the bond or interaction strengthening^68–70^. Multiwfn was used to study the character of BCP^71^. More attention are paid to the electron density ρ(r) and potential energy density V(r) at BCP between the hydrogen bond^72^.

## Results and Discussion

### Hydrogen Bonding Dynamics

**Figure 2:**
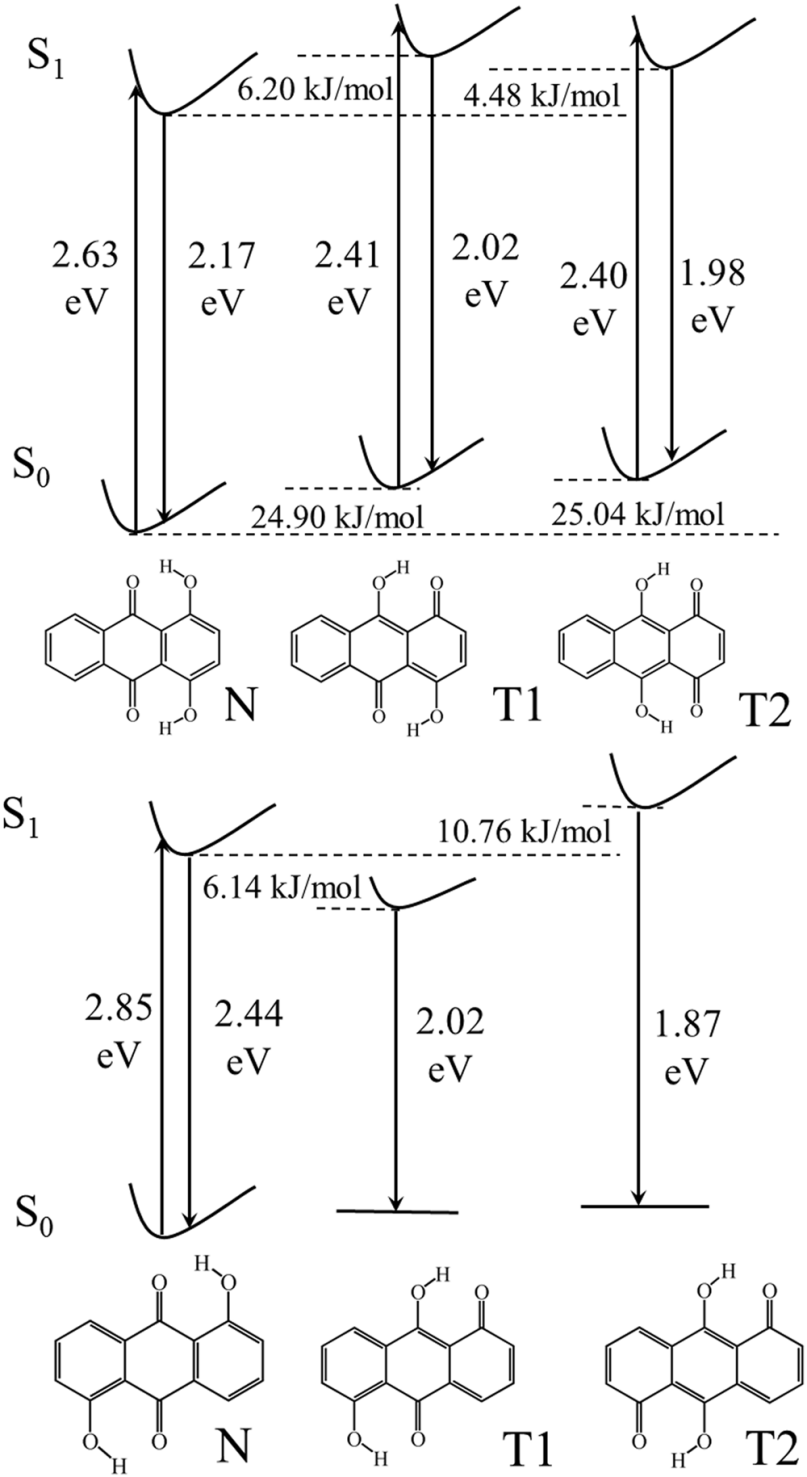
Stable structures of 1,4-DHAQ and 1,5-DHAQ in ground states and excited states for ESIPT tautomers.

Figure 1 shows the molecular structures for AQs studied in this work. The n-heptyl group is replaced by the methyl group, as to simplify the DFT calculations. In the optimized structures in the ground and excited states, the atoms involve in ESIPT progress are in the same plane with the benzene rings. The geometric parameters of eight compounds in the ground and excited states are listed in Table 1, as to illustrate the changes in the hydrogen bonding interactions upon photoexcitation. The calculated systems are not stable in ground states (S_0_(T)), except for 1,4-DHAQ whose stable structures are successfully obtained for both single proton and double protons transfer in ground state (Fig. 2). Comparison of the hydrogen bond lengths in ground and excited states showed that the hydrogen bonds enhanced in the sequence of 1-HAQ > 1,5-DHAQ > 1,8-DHAQ > 1,4-DHAQ. The hydrogen bond distances between donor O and H atoms in S_1_(N) and S_1_(T) are 1.008 Å and 1.459 Å for 1,8-DHAQ, and 1.014 Å and 1.540 Å for 1,4-DHAQ respectively, suggesting that the proton transfer of 1,4-DHAQ experienced a larger distance compared to 1,8-DHAQ. The changes in hydrogen bond distance of AYAAQs result from its electron-withdrawing ability. AAAQ did not present a stable structure for S_1_(T), consistent with the absence of LWE in the experiments. On the other hands, these molecules show the similar O_D_(N_D_)-H bond weakening tendency, suggesting that strengthening of the hydrogen bond promoted the ESIPT process.

**Table 1:** Bond length (Å) comparison of ground state and excited state hydrogen bond strength.

	Ground state		Excited state (N)		Excited state (T)	
	${{\bf{O}}}_{{\bf{A}}}\cdots {\bf{H}}$	O_D_(N_D_)-H	${{\bf{O}}}_{{\bf{A}}}\cdots {\bf{H}}$	O_D_(N_D_)-H	O_A_-H	${{\bf{O}}}_{{\bf{D}}}({{\bf{N}}}_{{\bf{D}}})\cdots {\bf{H}}$
1-HAQ	1.660	0.995	1.421	1.071	1.026	1.535
1,4-DHAQ	1.670	0.991	1.577	1.014	1.023	1.540
1,5-DHAQ	1.682	0.989	1.548	1.020	1.024	1.541
1,8-DHAQ	1.695	0.986	1.586	1.008	1.048	1.459
AAAQ	1.824	1.021	1.615	1.054	—	—
CAAQ	1.821	1.023	1.581	1.062	1.042	1.534
DCAQ	1.789	1.026	1.544	1.074	1.025	1.584
TFAQ	1.822	1.025	1.506	1.086	1.021	1.600

The role of dispersion interaction in hydrogen bonding interaction are considered by calculating the eight ground states structures with B3LYP-D3^73^. The data and comparison with B3LYP are listed in Table S1. The difference is negligible, which mostly result from the strong hydrogen bond in such systems. The B3LYP-D3 assuredly improved the result. While considering the minor differences and the comparion with excited state, we used the B3LYP for the calculation.

### Absorption/emission spectra and FMO analysis

The electronic excitation energies and corresponding fluorescence emission spectra of low-lying excited states for eight compounds were calculated by TDDFT method. The data are list in Table 2, which are consistent with the available experimental data. Generally, the energies of charge transfer states are underestimated in traditional functional compared with the long-range correction (LC) methods^74,75^. Then, we use the electron−hole analysis function to estimate the degree of charge transfer and calculate the vertical excitation energy with LC-BLYP. As shown in Tables S2 and S3, we think that except 1,5-DHAQ all other compounds have some portion of CT excitation. Compared with the B3LYP functional, the LC-BLYP functional significantly overestimate the excitation energy and show larger deviation with the experiment. While the B3LYP functional underestimates the energies, it give more accurate result. The molecules except for AAAQ are stable after proton transfer in excited state, with the different conformations. The relative energies of S_1_(T) were higher than S_1_(N) for 1,4-DHAQ, 1,8-DHAQ and CAAQ in ESIPT progress (Figs 2 and S1). 1,4-DHAQ and 1,5-DHAQ exist double ESIPT phenomenon with different properties in the ground and excited states (Fig. 2). 1,4-DHAQ and 1,5-DHAQ presented the S_1_ energies in the order of the T1 > T2 > N and T2 > N > T1, respectively. Thus, a fast ESIPT process is expected for 1,5-DHAQ and 1,4-DHAQ with the double ESIPT process. Furthermore, the energy of 1,8-DHAQ became slightly higher after ESIPT (Figure S1), giving rise to the solvent-sensitive ESIPT process for 1,8-DHAQ^25^.

**Table 2:** Comparison of experimental and calculated absorbance and fluorescence emission band at the TDDFT/B3LYP /6–311 + G (d, p) level (unit is eV).

	Electronic Absorption		Fluorescence Emission	
	Theoretical data	Experimental data^a^	Theoretical data	Experimental data^a^
1-HAQ	2.96	3.06	2.37/2.06	2.48/2.10
1,4-DHAQ	2.63	2.61	2.17/2.02	2.22
1,5-DHAQ	2.85	2.90	2.44/2.02	2.10
1,8-DHAQ	2.84	2.89	2.41/2.02	2.42/ 2.10
AAAQ^b^	2.83	3.00	2.30	—
CAAQ	2.89	3.15	2.34/1.87	2.43/1.95
DCAQ	2.94	3.18	2.34/1.88	2.42/1.95
TFAQ	2.97	3.24	2.39/1.91	1.91

^a^The experiment data was adapted from ref.^42^. ^b^The data used for AAAQ is HAAQ (1-heptanoylamino AQ) to simplify calculation.

The AYAAQs can be classified into three categories by their ESIPT proprieties (Figure S1). AAAQ failed to get a stable structure after ESIPT in the TDDFT calculation, consistent with the experiments. After ESIPT, the energy become higher for CAAQ and slightly lower for DCAQ, verifying the double emission in dichloromethane and acetonitrile. In TFAQ, ESIPT is an intense exothermic progress, leading to a “barrierless” reaction.

**Figure 3:**
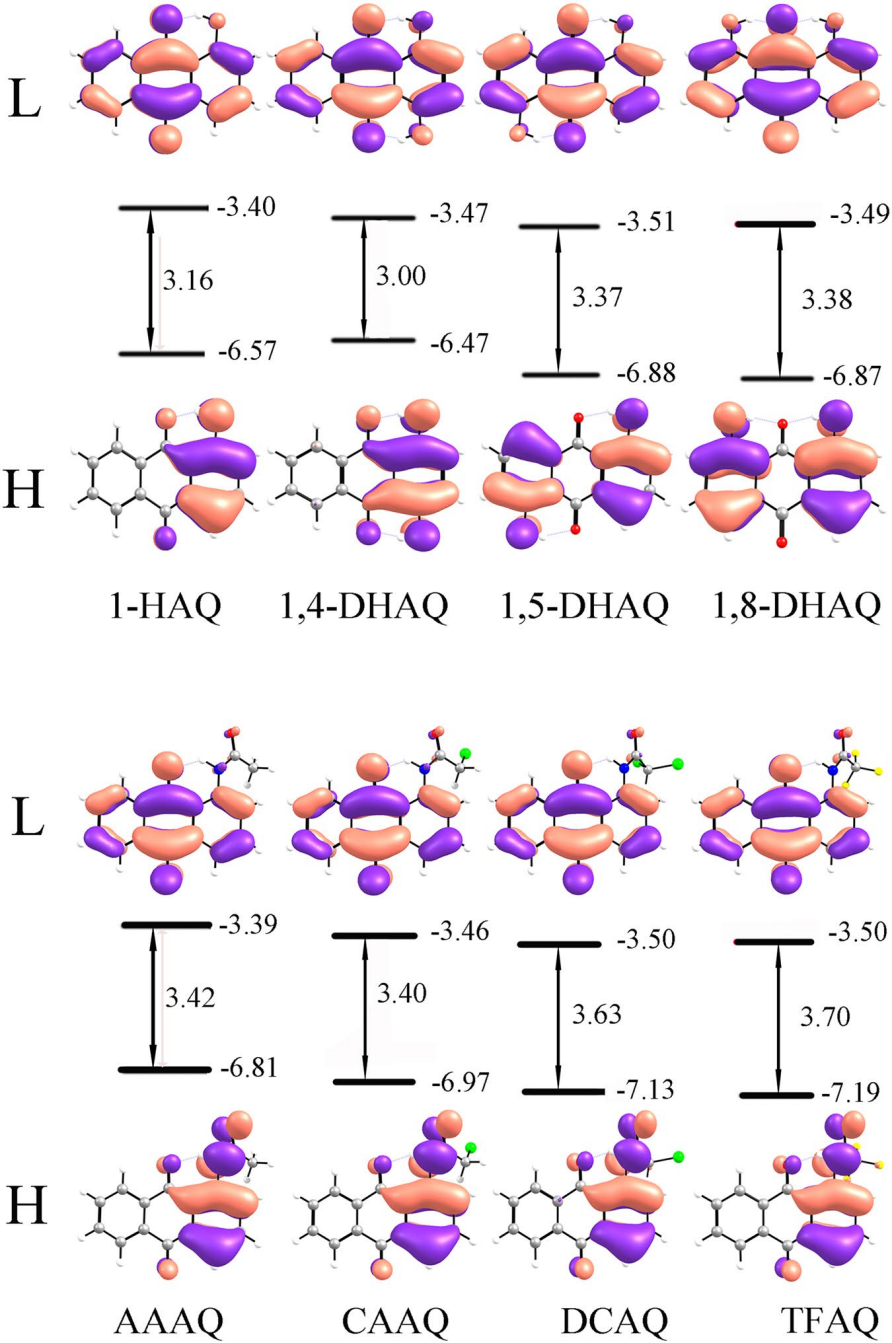
The schematic diagram of HOMOs and LUMOs for eight compounds and orbital energy levels (in eV).

Eight compounds have uneven HOMO distributions concentrating on the functional groups, and the evenly distributed LUMO (Fig. 3). The hydroxyl groups in different compounds show a stronger influence on HOMO compared to LUMO. The π-orbital can be observed at the carbonyl groups of 1-HAQ and 1,4-DHAQ in HOMO, while no distribution appeared in 1,5-DHAQ and 1,8-DHAQ. The 1,4-DHAQ has the a small energy gap, resulting in the longest absorption wavelength in all DHAQs^76^. The properties of compounds 1,5-DHAQ and 1,8-DHAQ are similar. The FMO plots of AYAAQs show the identical HOMO and LUMO, suggesting that their properties depend on the functional groups, in other words the electron-withdrawing ability. The energy levels of HOMO and LUMO decline from AAAQ to TFAQ, with the gap increased which is consistent with the fact that blue shift of the absorption wavelength in experiments^40,43^.

### Potential energy surface

**Figure 4:**
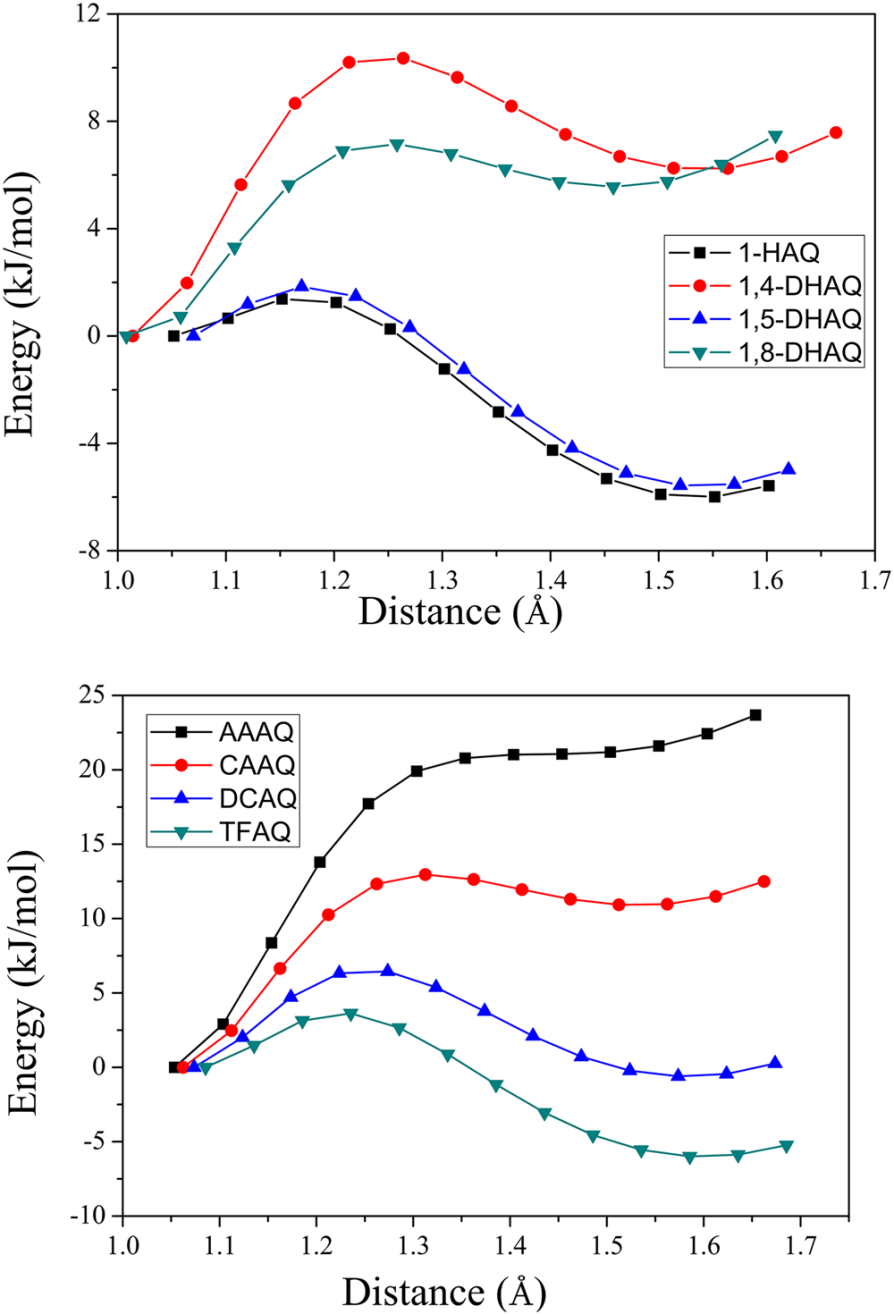
The scan of PES of S_1_ state of eight compounds as a function of O_D_(N_D_)–H bond length. The energy of stable structure in S_1_ state after structure optimization is set as zero point.

Potential energy surfaces (PESs) were scanned for molecules at S_0_ and S_1_ states. Figure 4 shows the PESs in S_1_ state for the eight compounds, while that in S_0_ state are showed in Figure S2. For HAQ and DHAQs, the energy barriers for ESIPT follow an order of 1,4-DHAQ > 1,8-DHAQ > 1,5-DHAQ ≈ 1-HAQ. The energy barrier is up to 10 kJ/mol for 1,4-DHAQ with the energy of S_1_(T) much higher than S_1_(N). 1-HAQ and 1,5-DHAQ exhibit the energy barriers lower than 2 kJ/mol, suggesting a “barrierless” ESIPT process. 1,4-DHAQ and 1,8-DHAQ have different O_D_-H bond lengths at S_1_(T), i.e., 1.540 Å and 1.459 Å for 1,4-DHAQ and 1,8-DHAQ, respectively. The shorter distance of proton transfer for 1,8-DHAQ is expected to be benificial for the ESIPT. The decreased energy barrier and enhanced exothermic effects from AAAQ to TFAQ are consistent with the experiments. CAAQ has the energy barrier of 13 kJ/mol, higher than DCAQ and TFAQ. Thus, the electron-withdrawing groups in proton donor part promote the ESIPT process by strengthening the hydrogen bond in excited state.

### Excited state hydrogen bonding dynamics and AIM analysis

**Figure 5:**
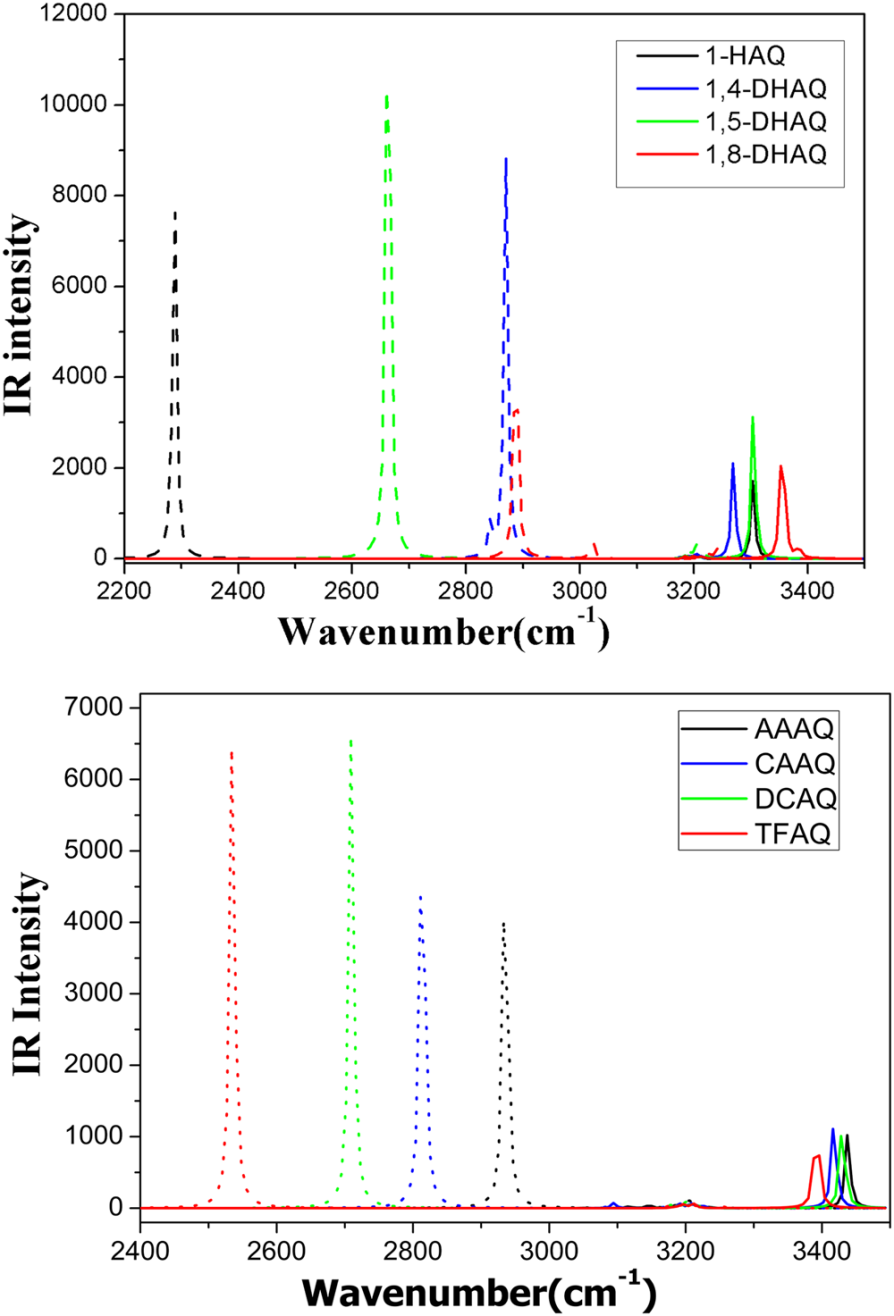
The calculated IR spectra of eight compounds in the spectral region of both O–H or N-H stretching modes in the S_0_ and S_1_ states (the solid line and dash line show the corresponding vibrational modes in S_0_ and S_1_ states, respectively).

The comparison in the vibrational frequencies of O–H or N-H stretching modes have been proved as a very reliable method to monitor the hydrogen bond strengthening and weakening from ground to s^15,21,77–79^. The calculated vibrational frequencies are presented in Fig. 5. The redshift of O–H or N-H stretching modes suggests the strengthening of hydrogen bond. For HAQ and DHAQs, the vibrational frequency is located at 3400–3600 cm^−1^ in ground state and widely disperse from 2200 to 3000 cm^−1^ in excited state. The DHAQs have two O–H stretching modes, i.e., anti-symmetry (*as*) mode and symmetry (*s*) mode, the intensity presents large difference and one mode are hardly distinguished from the IR spectra. Larger intensity O–H stretching in excited state appeared in *s* mode for 1,4-DHAQ and *as* mode for 1,5-DHAQ and 1,8-DHAQ. The largest shift is nearly 1000 cm^−1^ for 1-HAQ, suggesting a strongest hydrogen bond strengthening. The shift of 1,4-DHAQ and 1,8-DHAQ is relatively small, especially for 1,4-DHAQ without ESIPT process. The hydrogen bond of AYAAQs are strengthened to different extent. The N-H stretching mode densely distributed around 3400 cm^−1^ in ground state and dispersed form 2500 to 3000 cm^−1^ in excited state. The corresponding redshift is 548 cm^−1^ for AAAQ and 952 cm^−1^ for TFAQ. The sequence of strengthening is coincident with the degree and rate of ESIPT (TFAQ > DCAQ > CAAQ > AAAQ).

The bond critical point (BCP) generally appears between attractive atom pair. The value of real space functions at BCP have great significance to analyze the weak interaction^80–82^. For example, the value of ρ(r) at BCP is closely related to bond strengthening in analogous bond type, and the V(r) at BCP has been shown to be highly correlated with hydrogen bond energies. The relationship between hydrogen bond energy E_HB_ and V(r) at corresponding BCP can be approximately described as^72^:

$${{\rm{E}}}_{{\rm{HB}}}={\rm{V}}({\rm{r}})/2$$

The extent of the hydrogen bond strengthening after photoexcitation is calculated the change of ρ(r) and V(r) at BCP in ground states and excited states (Tables 3 and 4). Upon photoexcitation, all the hydrogen bonds are enhanced. Density of all electrons ρ(r) at BCP position show the similar tendency. For clear comparison, we define the change of ρ(r) and V(r) as Δρ% and ΔV% as Δρ% = (ρ_ES_ − ρ_GS_)/ρ_GS_ × 100% and ΔV% = (V_ES _− V_GS_)/V_GS × _100%. The three DHAQs have double hydroxyls while the calculated ρ(r) and V(r) are only characterized at single BCP. The molecules that present dominant ESIPT fluorescence emission in different solvents have a lager degree strengthening, such as 1-HAQ, 1,5-DHAQ, DCAQ and TFAQ and their ΔV% are higher than 104%. The 1,4-DHAQ and AAAQ which without ESIPT progress have lowest range of strengthening which the ΔV% are lower than 72%. Those with solvent-depended ESIPT progress have a moderate strengthening, such as CAAQ and 1,8-DHAQ. While the Δρ% of AAAQ and 1,8-DHAQ are same but with different properties. Thus, the ΔV% is a better reference than Δρ% when conjecture the property of ESIPT.

**Table 3:** Density of all electrons ρ(r) at BCP position.

	GS	ES	Δρ%
1-HAQ	132.85	253.99	92%
1,4-DHAQ	136.00	170.53	25%(51%)
1,5-DHAQ	132.06	184.05	39%(79%)
1,8-DHAQ	127.07	164.33	29%(59%)
AAAQ	93.78	154.93	65%
CAAQ	94.24	168.64	79%
DCAQ	87.51	185.28	112%
TFAQ	93.42	204.40	119%

**Table 4:** Potential energy density V(r) at BCP position.

	GS	ES	ΔV%
1-HAQ	−131.11	−289.07	120%
1,4-DHAQ	−135.21	−181.55	34%(69%)
1,5-DHAQ	−129.96	−200.48	54%(109%)
1,8-DHAQ	−125.29	−175.88	40%(81%)
AAAQ	−80.97	−136.58	69%
CAAQ	−81.18	−152.93	88%
DCAQ	−72.84	−174.41	142%
TFAQ	−79.55	−202.19	154%

## Conclusion

DFT/TDDFT methods were employed to investigate the ESIPT process of eight AQs compounds. By analyzing the geometric structures, absorption/fluorescence spectra, infrared vibration and AIM, the ultrafast ESIPT processes for eight molecules were systemically studied. Hydrogen bond strengthening in excited state verified by the decreased hydrogen bond lengths, redshift of O–H or N-H stretching vibration modes and the increase of ρ(r) and V(r) at BCP are the driving forces for proton transfer in the excited states. The electron-withdrawing groups play the role in strengthening the hydrogen bondof O_A_…H. The exothermic reaction and “barrierless” ESIPT process are observed for 1-HAQ, 1,5-DHAQ and TFAQ with the barrier lower than 2 kJ/mol. 1,4-DHAQ have the highest transfer barrier hindering the occurrence of ESIPT. The alterable ESIPT property of 1,8-DHAQ, DCAQ and CAAQ mainly owes to their medium barrier and similar energy of S_1_(N) and S_1_(T). The change of electrons density ρ(r) and potential energy density V(r) at BCP position in ground state and excited state are the important indicators for the ESIPT process. The V(r) at BCP position is the general reference for various kinds of hydrogen bonds.

## Electronic supplementary material

Supplementary Information
