# Supplementary material for: Combined TDDFT and AIM Insights into Photoinduced Excited State Intramolecular Proton Transfer (ESIPT) Mechanism in Hydroxyl- and Amino-Anthraquinone Solution
Source: Sci Rep. 2017 Oct 23;7:13766. doi: 10.1038/s41598-017-14094-5 (PMC5653788; doi:10.1038/s41598-017-14094-5)
Supplement: Supplementary file 1 — Supplementary Information [file 41598_2017_14094_MOESM1_ESM.docx]

**Supplementary Information to**

**Combined TDDFT and** **AIM Insights into Photoinduced Excited State Intramolecular Proton Transfer (ESIPT) Mechanism in Hydroxyl- and Amino-Anthraquinone Solution**

Daoyuan Zheng,^1,3^ Mingzhen Zhang,^1,2^ Guangjiu Zhao^1,2*^

*^1^State Key Laboratory of Molecular Reaction Dynamics, Dalian Institute of Chemical Physics, Chinese Academy of Sciences, Dalian 116023, China;*

^2^*Tianjin Key Laboratory of Molecular Optoelectronic Science, Institute of Chemistry, Department of Chemistry, School of Science, Tianjin University, Tianjin 300072, China;* ^3^*University of the Chinese Academy of Sciences, Chinese Academy of Sciences, Beijing 100049, China.*

**To whom correspondence should be addressed*

E-mail Address: gjzhao@tju.edu.cn

**Table S1**. The comparison of B3LYP and B3LYP-D3 in aspect of the root mean square deviation (RSMD), O-H vibrational frequency (Freq), ρ(r) and V(r) at BCP

|  | RSMD  (Å) | Freq (cm^-1^) | | ρ(r) (kJ/mol) | | V(r) (kJ/mol) | |
| --- | --- | --- | --- | --- | --- | --- | --- |
|  |  | B3LYP | B3LYP-D3 | B3LYP | B3LYP-D3 | B3LYP | B3LYP-D3 |
| 1-HAQ | 0.004 | 3304 | 3294 | 132.85 | 135.06 | -131.11 | -134.16 |
| 1,4-DHAQ | 0.003 | 3272(as)  3269(s) | 3258(as)  3256(s) | 136.00 | 138.63 | -135.21 | -138.81 |
| 1,5-DHAQ | 0.005 | 3305(as)  3302(s) | 3293(as)  3289(s) | 132.06 | 134.53 | -129.96 | -133.35 |
| 1,8-DHAQ | 0.005 | 3385(s)  3356(as) | 3375(s)  3345(as) | 127.07 | 129.31 | -125.29 | -128.36 |
| AAAQ | 0.030 | 3437 | 3442 | 93.78 | 94.78 | -80.97 | -82.26 |
| CAAQ | 0.037 | 3418 | 3424 | 94.24 | 94.96 | -81.18 | -82.13 |
| DCAQ | 0.037 | 3430 | 3438 | 87.51 | 87.77 | -72.84 | -73.20 |
| TFAQ | 0.032 | 3392 | 3398 | 93.42 | 94.36 | -79.55 | -80.73 |

**Table S2**. The calculated value of Δr and S in B3LYP/6-311+g(d)

|  | 1-HAQ | 1,4-DHAQ | 1,5-DHAQ | 1,8-DHAQ | AAAQ | CAAQ | DCAQ | TFAQ |
| --- | --- | --- | --- | --- | --- | --- | --- | --- |
| Δr/Å | 2.13 | 1.57 | 0 | 0.635 | 2.54 | 2.21 | 1.87 | 1.68 |
| S | 0.300 | 0.410 | 0.383 | 0.375 | 0.308 | 0.336 | 0.328 | 0.319 |

**Table S3**. Comparison of calculated of LC-BLYP and B3LYP with experiment excitation energy (unit is eV)

|  | LC-BLYP | B3LYP | Exp data |
| --- | --- | --- | --- |
| 1-HAQ | 3.76 | 2.96 | 3.06 |
| 1,4-DHAQ | 3.45 | 2.63 | 2.61 |
| 1,5-DHAQ | 3.86 | 2.85 | 2.90 |
| 1,8-DHAQ | 3.73 | 2.84 | 2.89 |
| AAAQ | 3.74 | 2.83 | 3.00 |
| CAAQ | 3.74 | 2.89 | 3.15 |
| DCAQ | 3.74 | 2.94 | 3.18 |
| TFAQ | 3.76 | 2.97 | 3.24 |
| Deviation | 0.52~0.96 | -0.27~0.02 |  |


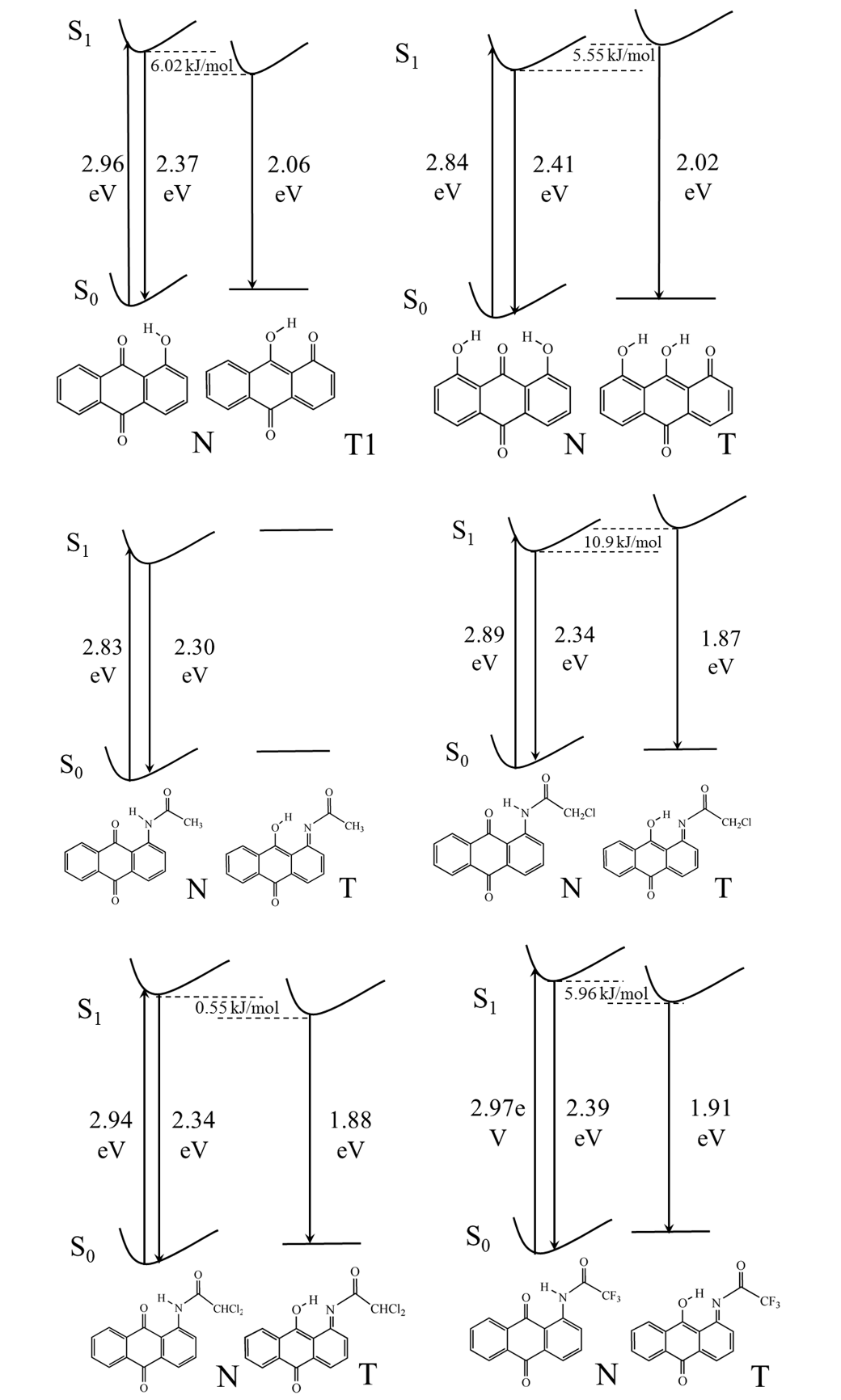


**Figure S1.** Relative energy for the critical structures of 1-HAQ, 1,8-DHAQ, AAAQ, CAAQ, DCAQ and TFAQ in ground states and excited states for ESIPT (the curve and straight line present with or without a stable structure, respectively)


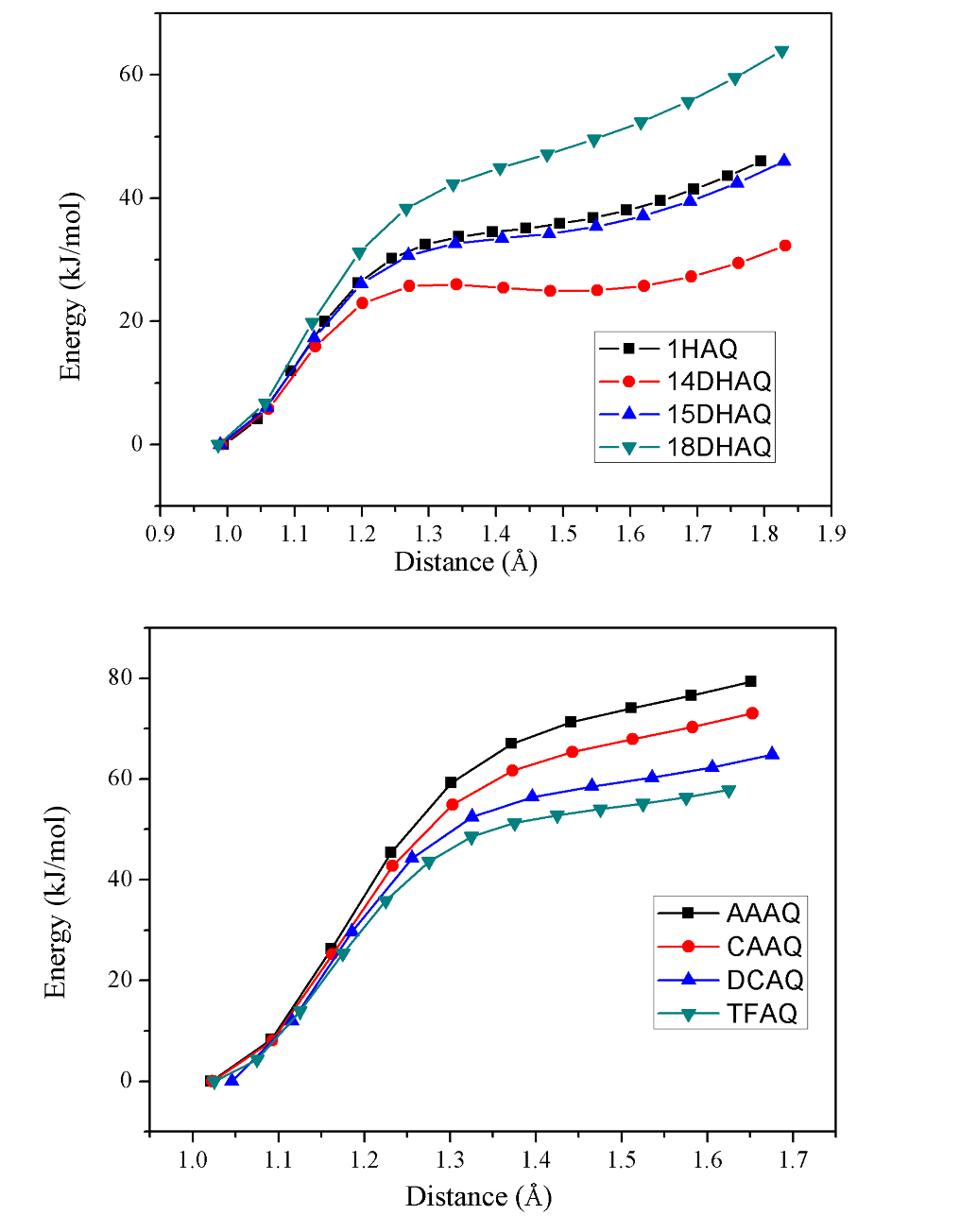


**Figure S2.** The scan of PES of S_0_ state of eight compounds as a function of O_D_(N_D_)–H bond length. The energy of stable structure in S_1_ state after structure optimization is set as zero point
